# Unveiling Atmospheric Layers: Vertical Pollution Patterns and Prospects for High-Resolution Aerosol Retrievals Using the Eastern Mediterranean as a Case Study

**DOI:** 10.1021/acs.est.4c14556

**Published:** 2025-06-10

**Authors:** Irina Rogozovsky, Albert Ansmann, Julian Hofer, Alexandra Chudnovsky

**Affiliations:** † Raymond and Beverly Sackler Faculty of Exact Sciences, Department of Geophysics, Air-O lab, 26745 Tel Aviv University, Tel Aviv 6997801, Israel; ‡ 28397 Leibniz Institute for Tropospheric Research, Leipzig 04318, Germany; § Raymond and Beverly Sackler Faculty of Exact Sciences, Department of Geophysics, Air-O lab, Tel Aviv University, Tel Aviv 6997801, Israel

**Keywords:** PollyXT lidar, MAIAC AOD, retrieval bias, AERONET, HYSPLIT, aerosol layering, random forest, pollution sources

## Abstract

The vertical distribution of aerosols plays a fundamental role in shaping air quality, influencing energy balance through radiative forcing, and impacting atmospheric dynamics. In the Eastern Mediterranean, we identify 10 distinct layering conditions characterized by specific vertical layering structures and aerosol mixing states. These configurations range from purely anthropogenic layers to complex multilayered mixtures, where marine aerosols, anthropogenic pollution, and dust occupy different altitudes, sometimes interacting or being further modified by rain. We conducted air mass back-trajectory clustering at 700 and 1700 m above ground level, linking pollution types to their transport origins. Satellite-derived aerosol optical depth biases were also systematically evaluated under various pollution scenarios, showing a strong satellite–ground correlations during dust events but poor accuracy under nondust conditions with marine–anthropogenic mixtures (35% of cases). Random Forest analyses demonstrated the potential to predict pollution layering types. Additionally, anthropogenic pollution content is increasing with altitude across all layering types, with evidence suggesting a growing prominence of anthropogenic pollution. These trends align with projections of a strengthening Persian Trough, the dominant summer synoptic system. This detailed categorization provides valuable insights into the complexity of pollution sources and atmospheric interactions in the region. The integration of vertical layering data holds significant potential, enhancing climate predictions, and pollution mitigation strategies on both local and global scales.

## Graphical abstract

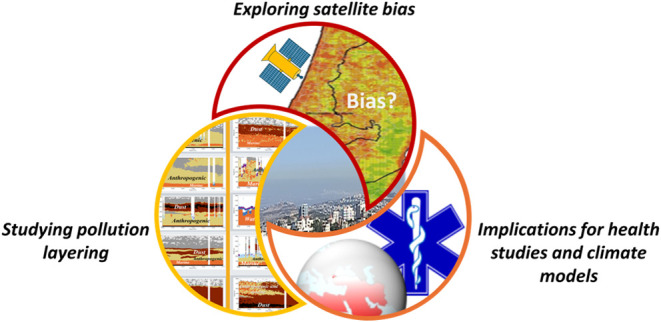

## 1 Introduction

Air pollution, based on the most recent estimation by the World Health Organization, and the Health Effects Institute & Institute for Health Metrics and Evaluation Global Burden of Disease study, contributes to 7 million and 8.1 million deaths per year, respectively.^1,2^ It remains one of the most pressing environmental health challenges, particularly in the Eastern Mediterranean (EM), where industrial activities, urbanization, and natural processes contribute to complex aerosol mixtures and layering structures.^3–7^ These layers are influenced by various sources of pollution, including mineral dust from Africa and the Middle East,^8^ industrial emissions from Europe and nearby coastal areas, marine aerosols from surrounding seas, biomass-burning smoke^9,10^ and local anthropogenic activities. The interaction and transport of these pollution sources create a highly dynamic and variable atmospheric environment,^11^ which is poorly understood in terms of its formation, movement, and predictability.

One of the key challenges in addressing air quality gaps lies in developing methodologies that account for the vertical distribution of pollutants and their complex interactions.^12^ Traditional approaches, which rely on surface-level measurements or columnar averages, often fail to capture the intricate layering and transport dynamics that influence regional air quality and climate. For example, conventional methods miss at least 50% of dust-contaminated days, resulting in significant underestimations in health impact studies and climate assessments.^13^ This limitation is particularly pronounced in regions such as the EM, where diverse atmospheric compositions complicate pollution assessments.^7^ Advanced methods that incorporate vertical pollutant distribution are therefore essential for improving air quality models and understanding pollution’s role in health and climate dynamics. To address these gaps, the vertical classification of pollution sources and their spatial variability emerges as a critical tool for accurate air quality monitoring.

Stratified layering conditions pose challenges for conventional satellite retrieval measures such as aerosol optical depth (AOD), which indicates overall columnar aerosol load and is frequently used to estimate surface-level fine particulate pollution concentrations. The retrieval accuracy is largely influenced by different layering conditions,^4,5^ with local sources concentrating in the boundary layer, while mineral dust or biomass-burning emissions often travel long distances and influence regional air quality in the upper layers.^14–16^ In view of the above, there is a need for refined methodologies to better represent these conditions.^4,5,17^

Lidar technology is essential for the vertical classification of air pollution, offering precise high-resolution measurements of aerosol layers and their sources at different altitudes by analyzing the properties of particle scattering and depolarization.^18–25^ Research has explored layering conditions, layer heights and impacts on urban air quality.^26–30^ Lidar has also been integrated with satellite-based aerosol retrievals and ground sensors to estimate 2.5 μm-diameter particulate matter (PM_2.5_).^31–36^ CALIOP (Cloud-Aerosol Lidar with Orthogonal Polarization) onboard the CALIPSO (Cloud-Aerosol Lidar and Infrared Pathfinder Satellite Observations) satellite further supports satellite–lidar validation.^4,5,37^ However, fine-scale vertical classifications remain poorly explored in urban regions, where local sources create complex pollution layers.

Our study emphasizes the importance of vertical classification in understanding air pollution dynamics in the EM region at high temporal resolution. The main goals are to (1) classify the vertical layering of daily atmospheric pollution conditions, (2) analyze their seasonal variations, and (3) assign each pollution type to its most representative air mass-transport pathway using back-trajectory analysis. As an example of future applications (4), we applied random forest (RF) analyses to assess the possibility of estimating each pollution layering condition using available ground-based and satellite measurements.

### 1.1 Novelty of the Study

- A systematic study of vertical layering structures and aerosol mixing states in the Eastern Mediterranean: Previous research has predominantly focused on individual case studies,^38–40^ and/or long-term aerosol typing.^6,41^ Our work analyses five years of high vertical (7.5 m) and temporal (30 s) resolution lidar observations, offering an unprecedented long-term assessment of vertical aerosol layering conditions, seasonal trends, meteorological influences, and the impact of regional synoptic systems in a climatically challenging environment.^42^
- Assessment of satellite aerosol optical depth (AOD) biases by pollution type: We provide the first detailed evaluation of how high-resolution 1 km AOD retrievals vary under different vertical pollution configurations, identifying conditions under which retrievals are biased and proposing improvements for future high-resolution satellite products.
- Seasonal and synoptic-driven pollution variability: Our study links synoptic-scale meteorology to vertical pollution structures, offering insights into how different atmospheric conditions shape the composition and altitude of pollution layers—an aspect often overlooked in previous studies.
- Integration of multiple data sets (e.g., AERONET, MAIAC, PollyXT, HYSPLIT): better characterization of different layering conditions and its further prediction.
- Future applications: Different aerosol layering configurations can be predicted and implemented in air quality forecasting and climate simulations.

## 2 Materials and Methods

The general methodology applied in our study consisted of the following main stages. First, we built a comprehensive data set to classify the different pollution layering types. For each type, we calculated its seasonal appearance and air mass back trajectories. Each type was tested for Multi-Angle Implementation of Atmospheric Correction (MAIAC) AOD bias by studying the correlation between MAIAC AOD and ground-based Aerosol Robotic Network (AERONET) AOD. In addition, using our unique data set, we identified the parameters that showed the strongest correlations with lidar-derived measurements for each type. Finally, as an example of future applications of our results, we present the results of a predictive model designed to classify each layering type using machine learning; here, we used a RF model.

### 2.1 Study Area

The Tel Aviv metropolitan area, located along the Mediterranean Sea, is home to approximately 4 million residents. It features a diverse urban landscape that includes parks, rivers, roads, and railways, as well as commercial and residential districts.

Israel’s seasonal synoptic conditions are shaped by six major synoptic groups driven by regional and global circulation Alpert et al.^43^ Winter features Cyprus lows, bringing intense rainfall,^43,44^ alongside Siberian and Subtropical highs. Summer is dominated by the Persian trough, causing stable weather. Red Sea trough can bring hot and dry conditions or, in certain cases, promote rainfall through moisture transport^45^ and Sharav lows drive hot and dusty winds in transitional seasons.

The lidar and AERONET stations used in this study are located at the Tel Aviv University (TAU) Faculty of Exact Sciences at 32.113° N, 34.806° E and an elevation of 76 m above sea level, ∼ 2.5 km from the shore (Figure S1).

### 2.2 Ground-Based Measurements

#### 2.2.1 PollyXT Lidar Observations and the Target Classification Product

The multiwavelength Raman polarization lidar PollyXT,^46^ located in Tel Aviv University (TAU), is part of PollyNET,^47^ operating continuously in 24 h a day/7 day a week mode. In this work, we used the lidar target classification product^48^ for the time period of September 2019–August 2024 at 18:00–19:00 UTC to classify the vertical layering of particles in the study area (total of 665 days).

The automatic generation of the target classification product is advantageous, ensuring efficiency and reproducibility. The classification product effectively distinguishes between a clear atmosphere (free of particles), clouds, and aerosols with high accuracy.^48^ Based on Baars method, aerosols are classified using the quasi particle depolarization ratio (*δp*) at 532 nm and the quasi Ångström exponent (AE) (532–1064 nm), which reflect the shape and size of the particles, respectively. Spherical particles (marine and anthropogenic) exhibit lower *δp* values, whereas nonspherical particles (dust) show higher values see Figure 7 and Table 1 in refs 49–51 . The AE indicates particle size, with higher values for smaller anthropogenic aerosols and lower values for larger natural aerosols, such as dust and marine particles.^52^ Based on these parameters, in the target classification product, aerosols are categorized into three main types (Figure 7 and Table 1 in Baars et al.^48^):

- Small aerosols (anthropogenic) – quasi AE ≥ 0.75 and quasi *δp <* 0.07
- Large spherical aerosols (marine/water droplets) – quasi AE < 0.75 and quasi *δp <* 0.07
- Large nonspherical aerosols (dust) – quasi *δp ≥* 0.07

The quasi *δp*, derived from the volume depolarization ratio and the quasi particle backscatter coefficient (with applied corrections and assumptions) (see eqs 8 and 10 in Baars et al.^48^), provides an approximation that closely aligns with the true particle backscatter coefficient and the depolarization ratio. The quasi AE is calculated using the quasi particle backscatter coefficients at 532 and 1064 nm (see eq 9 in Baars et al.^48^). The uncertainty in the particle depolarization ratio is 10%, while the particle backscatter uncertainty is 20%, resulting in a target classification error not exceeding 20%.^48^

#### 2.2.2 AERONET

For the measured AOD from the ground and the AE (440–675 nm), we used the AERONET located next to the PollyXT lidar at TAU. We used the AOD at all available wavelengths to interpolate the AOD at 470 nm (to align with the MAIAC AOD). We averaged the measurements closest to the 18:00–19:00 UTC time window to align with the lidar product’s measurement period (approximately 16 UTC). On days without AERONET measurements at TAU, we used AERONET data collected at the Weizmann Institute (located 27 km from the TAU AERONET). Based on our previous detailed analyses, these data are highly correlated with the TAU measurements.^5,13^ The data were downloaded from https://aeronet.gsfc.nasa.gov/ (last accessed 14 Aug 2024).

#### 2.2.3 Air Quality and Meteorological Data

In Israel, ground-monitoring stations measure mainly *PM*_2.5_ and *PM*_10_, referring to particles with aerodynamic diameters smaller than 2.5 and 10 μm, respectively. Similar to the AERONET measurements, we averaged the measurements closest to the 18:00–19:00 UTC time window to ensure consistency with the lidar product’s measurement period. The *PM*_10_ was obtained from the “University” environmental station, and the *PM*_2.5_ from the “New North”, both located in Tel Aviv. In addition, we used rainfall data, measured in millimeters, from the “University” station. Data were sourced from the Ministry of Environmental Protection’s Web site (https://www.air.sviva.gov.il//; last accessed 14 Aug 2024).

### 2.3 Satellite Data: Studying MAIAC AOD 1-Km Retrieval Bias

In this study, we used the daily averaged 1-km spatial resolution MAIAC AOD at 470 nm (MCD19A2 v006 data sets at https://lpdaac.usgs.gov/products/mcd19a2v061/; last accessed 27 Aug 2024), based on two retrievals for each day (Terra, ∼10:30 and Aqua, ∼13:30 equatorial crossing time).^53–55^ MAIAC AOD retrieval demonstrates relatively high accuracy in urban and high-reflectance regions (e.g., deserts)^56,57^ and under low-pollution conditions, closely aligning with ground-based measurements due to its fine 1-km spatial resolution.^58,59^ However, MAIAC’s performance decreases in environments with complex anthropogenic aerosol layers, as its algorithms are optimized for uniform or dust-dominated profiles. This limitation, evidenced by biases in mixed-layer pollution scenarios, indicates the need for further refinement to improve MAIAC accuracy.^4,5^

To deal with this limitation, we studied the MAIAC satellite AOD bias under different layering conditions. To that end, we compared the satellite-retrieved AOD with the AERONET AOD by examining the coefficient of determination for each layer type. AERONET AOD provides highly accurate, ground-based measurements of aerosol properties and considered a reliable reference due to its rigorous calibration and quality assurance protocols.^60^ Satellite AOD retrievals are generally compared to AERONET AOD as they help identify any biases or errors in satellite measurements.^4,5,61–68^ The strong correlation between Lidar AOD and AERONET AOD (*R* ^2^ = 0.95)^69^ indicating high consistency between the two measurement methods. By examining discrepancies between MAIAC and AERONET AOD across different aerosol layering types, we can identify systematic errors in MAIAC’s performance under specific atmospheric conditions.

We used AERONET AOD averaged to the closest available time of lidar measurements (see further explanation in Section 2.5). To account for the temporal mismatch between satellite overpasses and lidar observations, we incorporated daily averaged AERONET AOD to capture broader aerosol trends throughout the day. This approach helps to bridge the temporal gap and provides a more representative comparison between MAIAC AOD and lidar-derived parameters.

### 2.4 Vertical Layering Classification: Decision Tree

Figure S2 shows a decision tree for classifying different layering aerosol structures (based on target classification product, part of PollyXT retrieval^48^). Days were first categorized based on dust presence: if dust contributed less than 15%, the days were classified as dust-influenced, with subsequent categorization based on marine and anthropogenic contributions to identify either dominant aerosol types or complex layering structures. Each of the 665 days (September 2019–August 2024) was assigned to one of the 10 most prominent layering types (A–J; Figure 1) followed by manual verification of the classification. This systematic approach ensures a rigorous differentiation of aerosol layers based on their sources and vertical structure.

**Figure 1:**
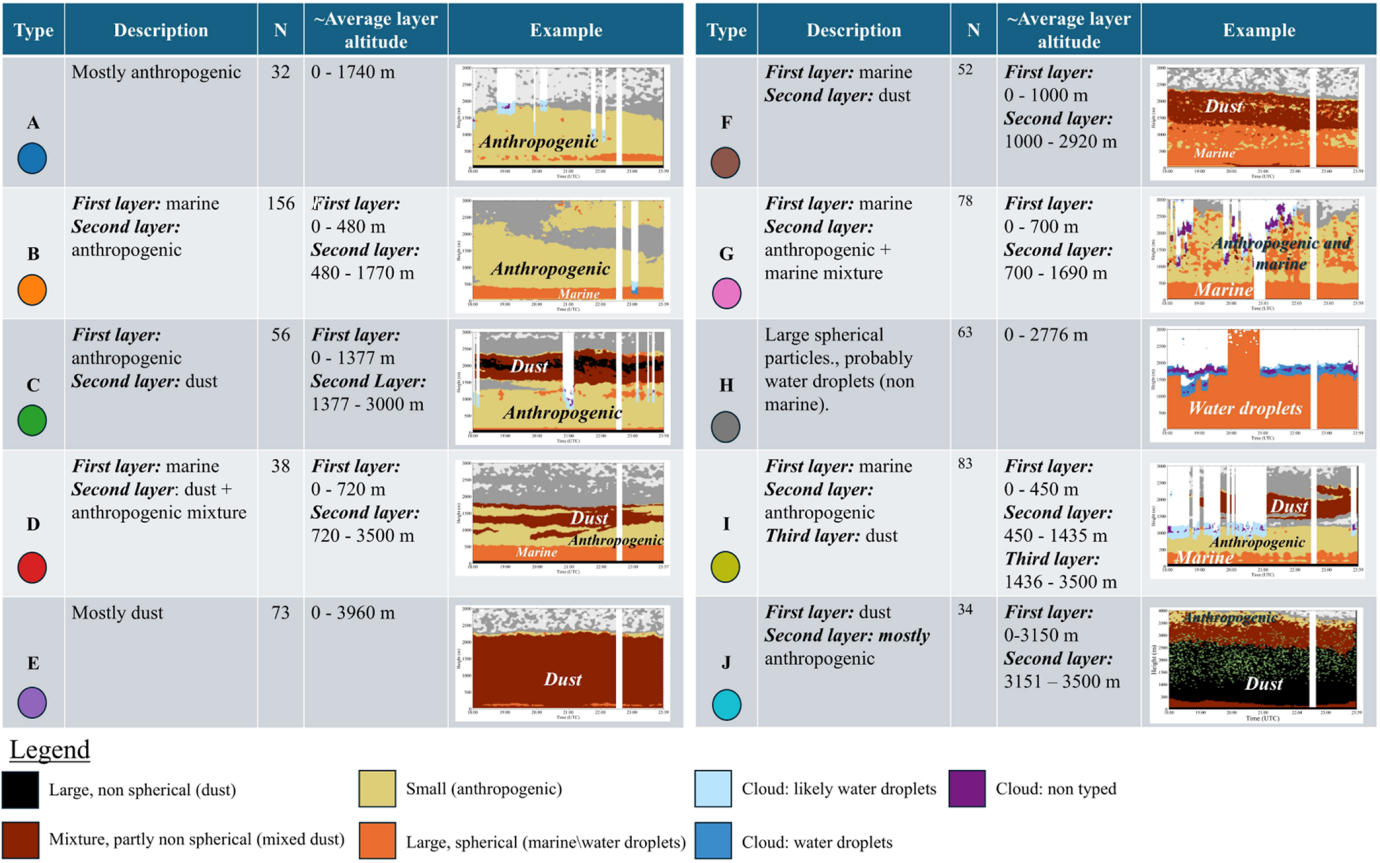
Classification of vertical layering types using the complete data set (2019–2024). The classification is based on the “target classification” product, which use quasi Ångström exponent (AE) and quasi depolarization ratio (*δp*) to categorize aerosols: Small aerosols (anthropogenic) – quasi AE ≥ 0.75 and quasi *δp <* 0.07. Large spherical aerosols (marine/water droplets) – quasi AE < 0.75 and quasi *δp <* 0.07. Large nonspherical aerosols (dust) – quasi *δp ≥* 0.07.^48^ Each type label (A-J) is accompanied by a distinct color, which will be consistently used to represent the corresponding type in all subsequent plots.

Finally, we compared PollyXT lidar ground-based measurements with CALIOP satellite retrievals, for two selected cases: homogeneous dust layering conditions (layering type E) and a complex pollution layering scenario (layering type B). Here, we aimed to examine how each instrument detects vertical aerosol structures CALIPSO Level 2 Vertical Feature Mask (VFM) product, version 4.5.1 (CAL_LID_L2_VFM-Standard-V4–51).^70^

#### 2.4.1 Optimal Timing: Nighttime Observations as a Representative Atmospheric Baseline

Similarly to Hofer et al.,^69^ we used the night observation window for the classification of the aerosol layers. Specifically, 18:00–19:00 UTC (If a target classification product for this specific time was unavailable, the file closest in time was used). The time was chosen for the following reasons: (1) Nighttime measurements benefit from the absence of sunlight, which reduces background noise and enhances the signal-to-noise ratio in lidar measurements.^71,72^ (2) During the daytime, the PBL evolves dynamically, growing and mixing pollutants vertically. At night, the PBL stabilizes,^73–75^ and the aerosol layers remain more stratified. This stability allows analysis of pollution transport and layering. (3) The impact of sea breeze is not dominant.^75,76^ Stagnant conditions improve the stratification of aerosol layers, providing a clearer understanding of the source contributions.

### 2.5 Layering Type Characterization: Merging Different Data Sets

By integration of multiple data sets (e.g., AERONET, MAIAC, PollyXT) we were able to find the most correlative parameters, to investigate the distribution of all measurements during different layering conditions, as well as to find the most significant variables for pollution types prediction. First, for each layering type, we averaged the following variables: lidar-derived parameters for the 0–1000 m and 1000–3000 m altitude ranges, including quasi AE (532–1064 nm), quasi-δ_*p*_ (532 nm), β (532 nm), and LR (532 nm). Furthermore, we incorporated ground-based and satellite-derived parameters: *PM*_10_, *PM*_2.5_ (close to the 18–19 UTC, Section 2.2.3), MAIAC AOD (daily average, Section 2.3), AERONET AOD (close to the 18–19 UTC, approximately 16 UTC) and AERONET AE (440–675 nm) (Section 2.2.2). The merging of different data sets was done using Python (version 3.12.3), the Pandas library for data integration.

We analyzed the relationship between quasi AE and quasi *δp* across different layering types, as these two variables serving as the basis for target classification. We analyzed AOD and AE values to distinguish aerosol types (with higher AE indicating fine-mode anthropogenic aerosols and lower AE signifying coarse-mode dust particles). Furthermore, *PM*_10_ and *PM*_2.5_ concentrations provide insights into near-surface aerosol loading. Specifically, dust layers near the ground elevate *PM*_10_ concentrations, while lofted dust layers have minimal surface impact.^13^ By integrating multiple data sets we validates our classification and ensures that it reflects real atmospheric conditions.

For each pollution type, we performed cross-correlation analyses between lidar-derived aerosol properties (*N* = 8) and nonlidar-derived measurements (*N* = 5) to assess their interrelationships. First, we quantified the significant correlations for each type of layering, allowing comparisons and identification of unique associations. Next, we identified the best-correlated properties for all layering types and each type individually. In addition, we study the seasonal distribution of each layering type.

### 2.6 HYSPLIT Back-Trajectory Analysis

The hybrid single-particle Lagrangian integrated trajectory (HYSPLIT) model is a tool developed by the National Oceanic and Atmospheric Administration (NOAA) to simulate air mass trajectories. It integrates meteorological data with particle physics to predict the movement of these substances.^77,78^ We used the HYSPLIT model to cluster 48-h back trajectories for air masses at 700 and 1700 m above ground level for each layering type. We tried different numbers of clusters—between 5 and 10—and ultimately chose 7 clusters because this number best represented the variety of regions from which the air masses came. This approach allowed us to study the most common source of air masses for each layering type.

### 2.7 Future Applications: Predicting Pollution-Layering Type

We studied the potential of machine learning techniques to predict vertical pollution layering types, as a preliminary groundwork for developing more precise air quality forecasting models. To that end, we applied random forests (RF), an ensemble learning method built on decision trees. RF is particularly suitable for environmental data, such as pollution levels, which are influenced by a complex interaction of meteorological, geographical, and anthropogenic factors.^79^ One key reason for choosing RF is its ability to handle high-dimensional and heterogeneous data sets, such as satellite, meteorological, and ground-based observations. Moreover, RF models are capable of managing missing values effectively and remain robust to measurement errors, a common characteristic in air quality data.^79^ Previous studies have established that RF excels in air quality classification and forecasting tasks, detecting intricate patterns in pollution concentrations that vary with seasonal changes or weather conditions.^80–87^

To predict vertical pollution layering types, we selected key input variables that represent both vertical and horizontal variability across different altitudes. To ensure that only the most relevant predictors were included in the model, we conducted a variable importance analysis (Mean Decrease Impurity^88^). This analysis enabled us to assess the relative contribution of each variable to the prediction of pollution layering types. Based on the variable importance scores, we selected the seven most significant variables, which delivered the highest performance in the model. These selected variables were then incorporated into the final RF model:

Lidar Ratio at 0–1000 m and 1000–3000 m. The lidar ratio helps estimate the scattering and absorption properties of aerosols, which are critical for understanding how pollutants behave in different layers of the atmosphere.

Quasi AE (Angstrom Exponent) at 0–1000 m, and 0–3000 m to get an insight into the size distribution of aerosol particles, which directly affects the pollution layering stratification.

Quasi *δp* at the same altitude ranges: 0–1000 m, and 0–3000 m, as an additional metric for aerosol shape and their distribution in the atmosphere.

Main Source of Air Masses: HYSPLIT back-trajectory analysis reveals that air masses reaching the study area originate from Africa, the Middle East, Europe, Asia, or marine regions. Identifying these sources at different altitudes is important for predicting pollutant types and understanding pollution transport dynamics.

## 3 Results and Discussion

### 3.1 Lidar-Based Classification

Figure 1 presents the common vertical layering structures of atmospheric pollutants, identifying 10 distinct types. These range from mostly anthropogenic pollution (Type A) to complex mixtures, such as layers of marine air overlain by anthropogenic or dust layers (Types B, F, G). Some configurations feature dust as the dominant pollutant, alone (Type E) or combined with anthropogenic pollution and marine aerosols (Types C, D, I, J). Other structures, such as Type H, indicate the presence of rain, which influences the layering of the pollution. These detailed classifications highlight the complexity of atmospheric layering in the EM, demonstrating that a detailed understanding of pollution dynamics is essential for air quality assessments.

The most common layering type was Type B (Figure 1), with a first (relatively thin) marine layer, and a (thicker) anthropogenic layer above it; 156 days out of the total 665 days (23%) were classified as Type B. This pattern may be linked to the specific conditions of coastal cities, where the sea breeze influence plays a key role, highlighting the impact of coastal proximity on aerosol dynamics.^75^

Ground-based measurements further support our aerosol classification, as presented in Table S1. For example: dust-dominant layering types E and J exhibit the highest mean *PM*_10_ concentrations (67.8 and 66.5 μg/m^3^, respectively), followed by layering type F (37.6 μg/m^3^), which also contains a significant dust component. As expected, AE values are highest for anthropogenic-dominant layering types (A, B, and G). Layering type E, representing pure dust, shows the lowest AE (0.46), while layering type J, which includes dust with anthropogenic contributions, exhibits a slightly higher AE of 0.92.

Figure 2 presents the comparison of type E and type B case studies as retrieved by PollyXT lidar vs CALIPSO. Both methods show general agreement in vertical structures for a dust and complex pollution cases over the entire region. However, CALIPSO’s significantly lower temporal resolution (single time shot rather than diurnal measurements) and coarser vertical resolution (30 m vs 7 m for PollyXT lidar) limit its applications and complex layering conditions monitoring.^89^

**Figure 2:**
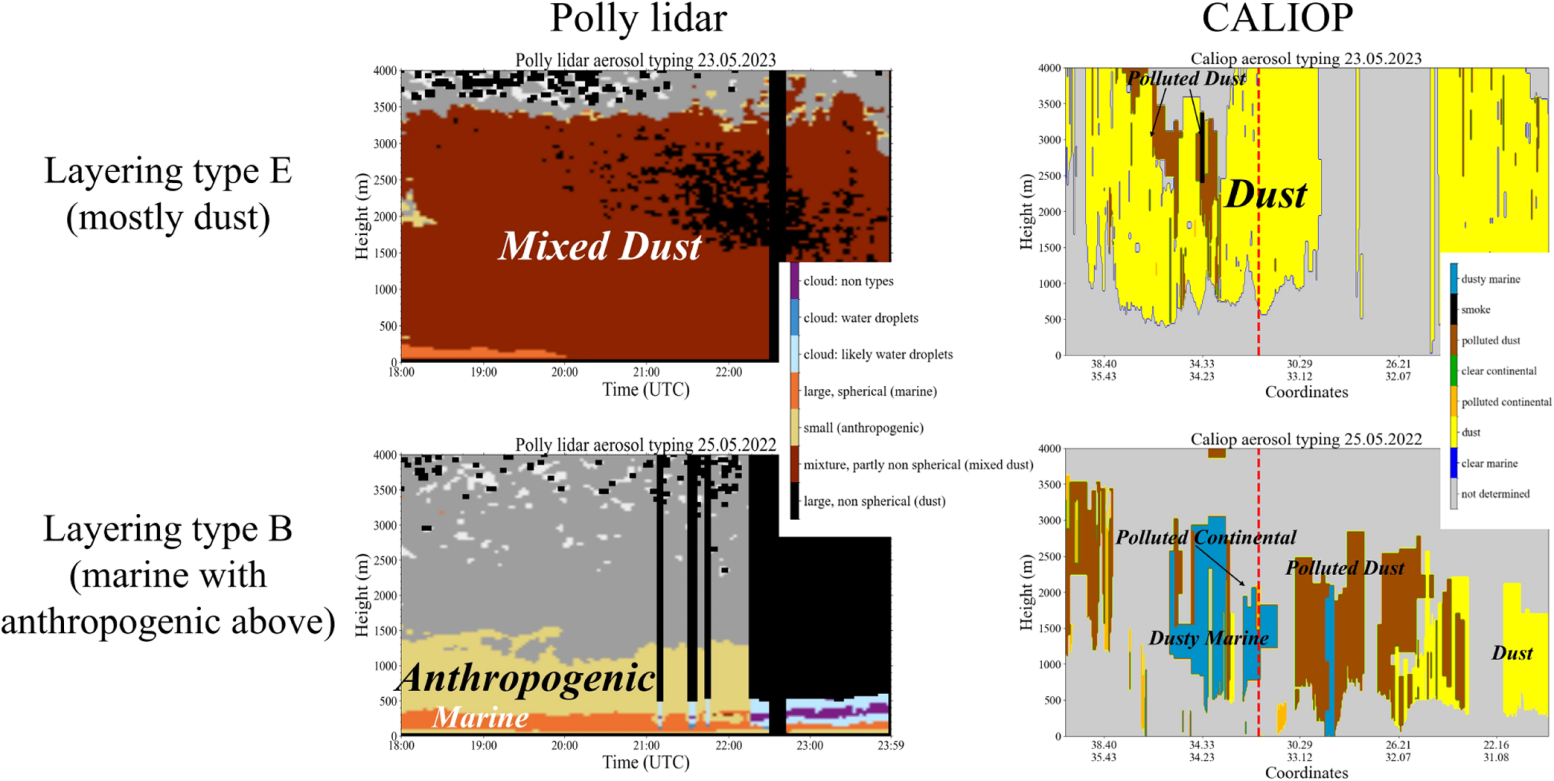
Comparison of aerosol typing from CALIOP (right) and PollyXT lidar (left) for two selected cases. The top row represents Layering Type E, dominated by dust, while the bottom row represents Layering Type B, characterized by a marine layer with anthropogenic aerosols above. CALIOP aerosol typing is derived from satellite-based observations along the track, where the *x*-axis represents geographic coordinates (the study area is marked by a red dashed line). In contrast, PollyXT lidar provides continuous ground-based observations at a fixed location, with the *x*-axis representing time.

Figure 3 presents the relationship between quasi AE (532–1064 nm) and quasi *δp* (532 nm) for heights of 0–1000 and 1000–3000 m. We marked the thresholds for dust, marine, and anthropogenic aerosols^48^ on the scatter plots with dashed red lines. In the lower layer, atmospheric conditions were generally dominated by marine particles, reflected in lower quasi AE values. In contrast, for 1000–3000 m, there was a notable increase in anthropogenic pollution, as indicated by higher quasi AE values across all layering types. Types J and E were dominated by dust aerosols, characterized by high quasi *δp* values. However, even within these dust layers, a stronger anthropogenic impact was observed in the upper atmospheric levels.

**Figure 3:**
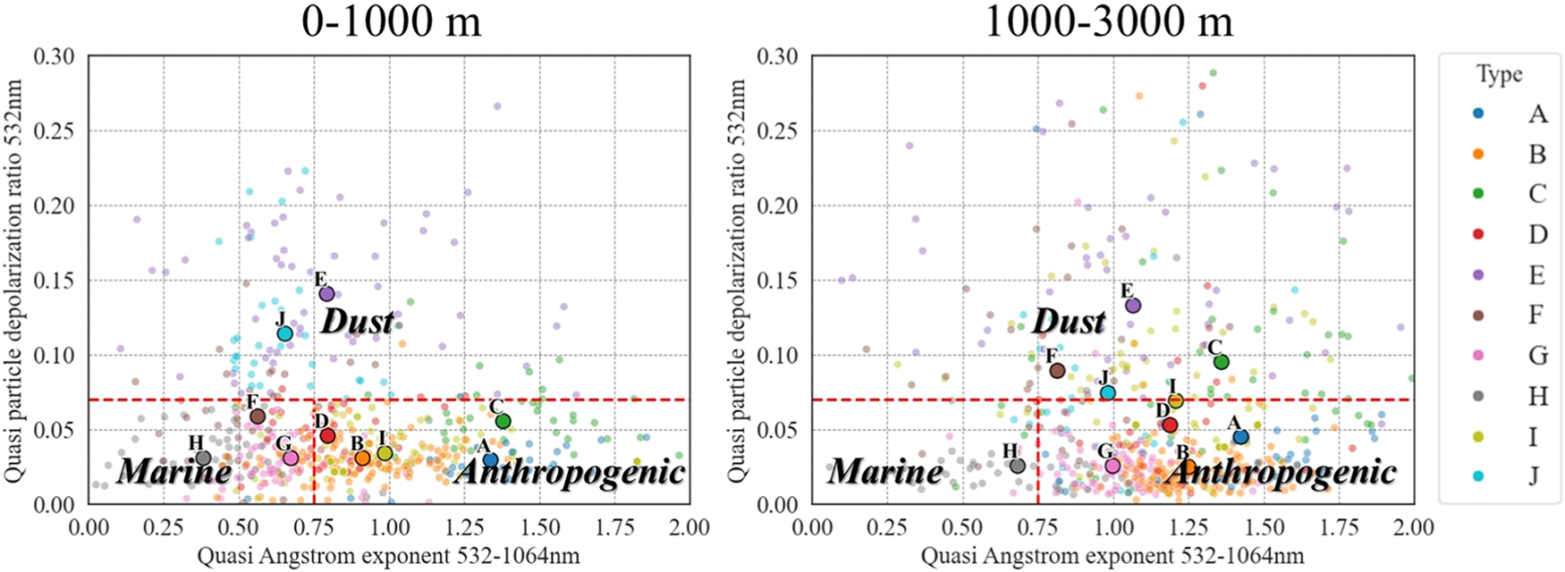
Scatter plots of the relationship between quasi AE (532–1064 nm) and quasi *δp* (532 nm) across three height ranges: 0–1000 m, and 1000–3000 m. Each point represents a specific day, categorized by type. The large points denote the average of each category from Figure 1. The dashed red lines highlight quasi AE and quasi *δp* thresholds as defined in Baars et al.^48^ to identify dust, marine, and anthropogenic aerosols. Note that most layering types move from the left side (low AE at height 0–1000 m) to the right (high AE, at height 1000–3000 m), indicating an increase in anthropogenic content with altitude.

Table S1 summarizes the β values for each layering type at different heights. Layering types E and H exhibit significantly higher β in the 1000–3000 m range. For all other layering types, β is higher in the lower layer (0–1000 m), which may indicate denser concentrations of particles closer to the surface.

### 3.2 Seasonal Variations of Aerosol Layering Conditions

Figure 4 shows the bar graph of the normalized frequency of each layering type on a seasonal basis.

**Figure 4:**
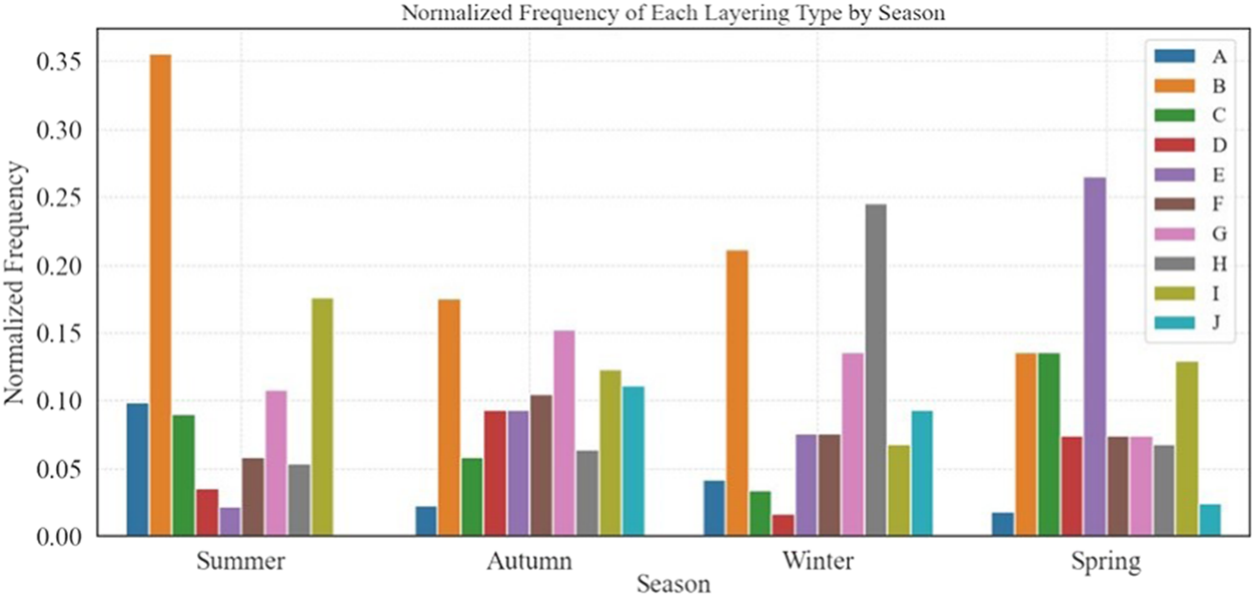
Bar plot of the normalized frequency of each layering type by season.

In the summer, anthropogenic aerosol layering dominates, with Type B and Type I (marine base and anthropogenic and dust) being the most prevalent. In particular, Type I includes lofted dust, challenging the assumption of dust-free summer conditions.^13,90^ Type A, which is mainly anthropogenic, also peaks in the summer (although not prevalent in this season), underscoring the anthropogenic influence in the EM.^42,91^ The combination of persistent westerly winds, occasional southerly dust transport, and stable summer conditions results in frequent Type B, Type I, and Type G layering. The dominant Persian trough winds bring marine air and transport anthropogenic pollutants eastward (e.g., coastal emissions mix with marine aerosols under stable conditions) and Type B (Marine + Anthropogenic) forms. Type G is formed by weak summer turbulence that allows some mixing but maintains stratification. For Type I, southerly winds from the occasional Red Sea Trough lift desert dust over the region (Marine + Anthropogenic + Dust Above).

The diversity of synoptic conditions in autumn—ranging from weakened summer troughs to early winter low-pressure systems—results in a wide range of pollution layering types (Figure 4). This seasonal variability explains why all pollution types are observed although Type B and Type G are dominant. The frequent occurrence of Type B (Marine + Anthropogenic) and Type G (Marine + Anthropogenic + Marine Mixture) reflects the transition between summer and winter atmospheric conditions. Low winds dominate and favor accumulation of local pollutions which combine with aged pollutions from southeast Europe and marine particles from the eastern Mediterranean Sea.

In spring, Type E (Dust-Dominated Layering) is the most frequent due to the seasonal dominance of Sharav Lows (Khamsin events) and the deepening of the Red Sea Trough. These synoptic systems generate strong south and southwest winds, transporting large amounts of dust from the Sahara Desert and the Arabian Peninsula to the eastern Mediterranean. Spring is characterized by dust transport at mid-to-high altitudes, leading to a distinct dust layer above the boundary layer.^92^

In the winter, the Cyprus Lows^43,93^ are the dominant synoptic systems, driving air mass transport primarily from the southwest to the northwest, and, following cold front passages, from the north. This circulation facilitates the advection of polluted air masses from North Africa, Europe, Russia, and Turkey to Israel. In addition, a persistent marine component is observed within the lowest 500–1000 m, corresponding to the typical height of the marine boundary layer, particularly in coastal regions. During frontal passages, Tel Aviv is influenced by humid air masses. As a result, layer types B (Marine + Anthropogenic) and H (Marine/Water droplets, rain) are dominant in the winter.

Over time, as evident from our analyses, the summer season shows an increase in types A (summer, anthropogenic), B (all seasons, anthropogenic), and C (spring, summer-anthropogenic+dust), particularly in 2024, while types D, E, and G decline. Winter layers remain relatively stable, though type H decreases. Despite these observed changes, the limited time frame is insufficient to draw definitive conclusions about long-term trends, emphasizing the need for extended monitoring.

### 3.3 The Major Sources of Air Pollution Transport: Back-Trajectory Analysis

The main air mass trajectories for the EM are Europe, the Mediterranean Basin, northern Africa, and the Middle East.^42^ Figure 5 shows air mass back-trajectory clustering results at heights of 700 and 1700 m above ground level for each layering type, with several key findings:

- Middle Eastern dust sources exhibit a higher anthropogenic component than North African dust.^94^ Type J (dust with an anthropogenic layer above) is consistently predominantly associated with Middle Eastern dust (74% at 700 m, 68% at 1700 m), whereas Type E, characterized by mostly pure dust, is associated with North African dust (51% at 700 m and 77% at 1700 m).
- Height-based differences: Types C, D, F, and I exhibit clear height-based variations, with marine and European air masses dominating at 700 m, and Middle Eastern or North African dust prevailing at 1700 m. In contrast, Types A, B, and G show consistent sources across altitudes, primarily marine air masses originating from Europe and Turkey, which collect marine particles at 700 m.
- Lofted dust layer: Types C, D, F, and I feature a lofted dust layer, with sources transitioning from marine and European origins at 700 m to North African and Middle Eastern dust at 1700 m.
- Type H primarily originates from southern Greece (33% at 700 m and 38% at 1700 m), with 10% from the Adriatic Sea at both altitudes and minor contributions from Scandinavia at 1700 m. These trajectories align with Cyprus lows, which are known to transport air masses associated with precipitation. This supports the classification of Type H as being linked to rain-associated water droplets.^44,95^

**Figure 5:**
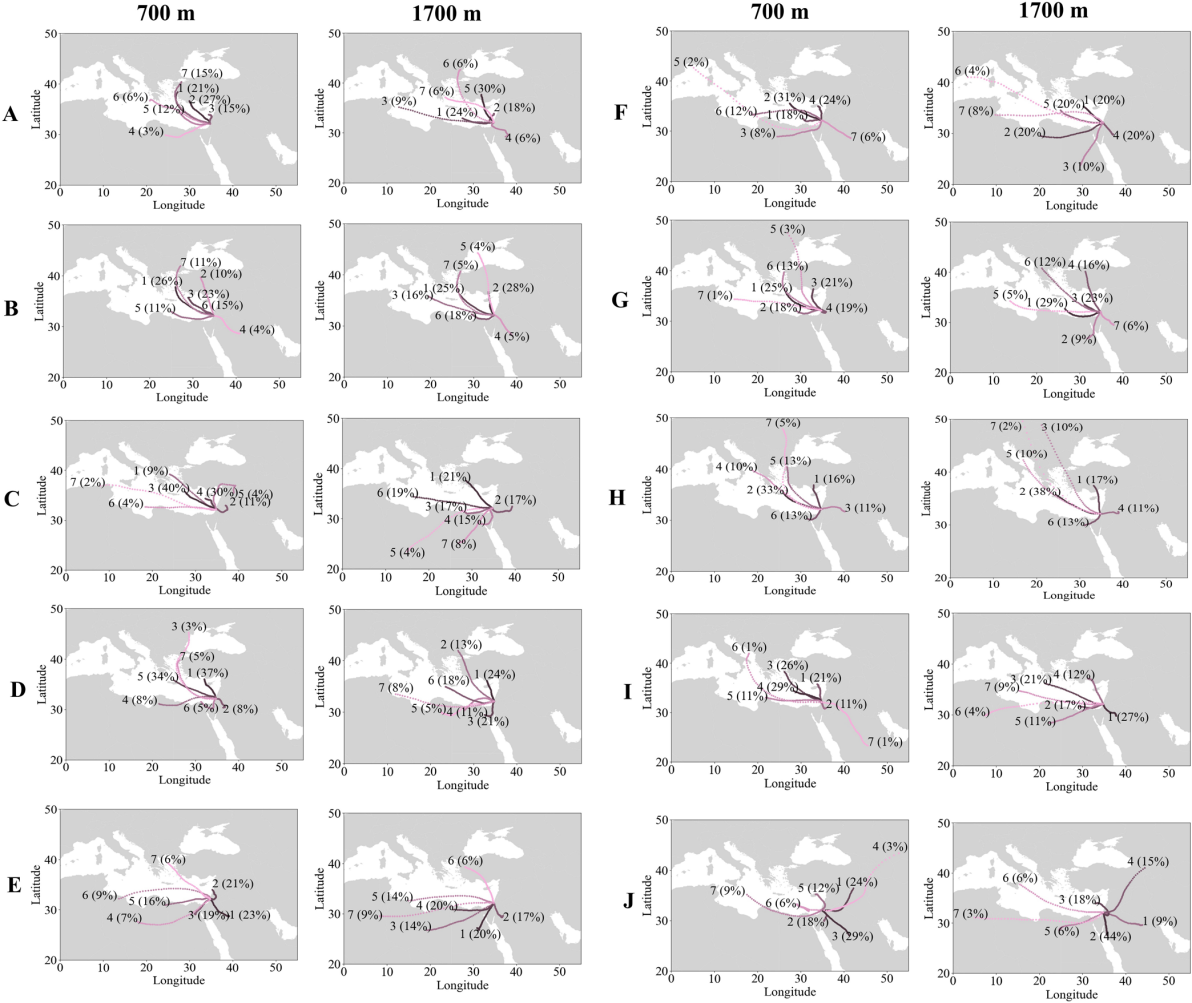
HYSPLIT cluster analysis of air mass back trajectories at 700 and 1700 m above ground level. Each line represents a trajectory cluster, with cluster numbers and frequencies indicated. Line colors denote frequency: darker shades correspond to higher frequencies, lighter to lower.

The results of our back-trajectory analyses align with the vertical layering patterns observed from lidar measurements (Figure 1), where marine and anthropogenic aerosols were prevalent in the lower layers during the summer and dust dominated at higher altitudes, whereas in spring, dust is transported from North Africa and in winter and autumn from the Middle East (Figure 4).

### 3.4 Satellite MAIAC AOD Bias Conditioned on Vertical Layering Type

Biases in MAIAC AOD retrievals have been observed in 30% of cases.^4^ Understanding the vertical classification of aerosol layers provides insight into the factors contributing to these biases, enabling more accurate interpretation of the retrievals. By analyzing correlations between MAIAC and AERONET AOD conditioned on aerosol layering types, we can identify systematic errors in MAIAC’s performance under specific atmospheric conditions. For instance, as shown in Figure 6, nondust layering types such as Type B (marine lower, anthropogenic upper) and Type G (marine lower, mixed upper) with low satellite–ground agreement (*R* ^2^ = 0.1 and *R* ^2^ = 0.01, respectively) accounted for 35% of the cases, highlighting significant retrieval challenges.

**Figure 6:**
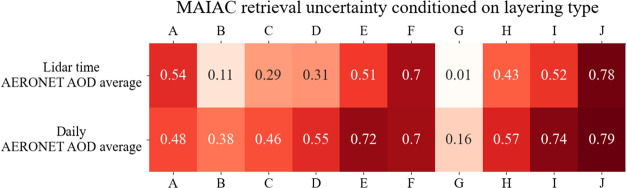
MAIAC retrieval uncertainty conditioned on layering type: coefficient of determination (*R* ^2^) values for the correlation between MAIAC and AERONET AOD (*R* ^2^ > 0.4 is considered a good correlation). The AERONET AOD was averaged for the time closest to the lidar observations (left) and daily averaged (right). Color scale represents *R* ^2^, ranging from bright colors for the lowest values to dark colors for the highest values.

In contrast, layering types influenced by Middle Eastern dust (Types F and J) demonstrated the best satellite–ground agreement (*R* ^2^ = 0.7 and *R* ^2^ = 0.78, respectively), likely due to MAIAC’s dust-related assumptions (e.g., contributions of coarse particles (dust) and some anthropogenic components); Model 2 in the MAIAC AOD retrieval^55^ aligns closely with these dust characteristics. Dust originating from North Africa (Type E) showed a moderate *R* ^2^ of 0.5, reflecting variability in particle properties.

While the daily averaged AERONET AOD demonstrates a stronger correlation with MAIAC AOD across all layering types, the overall trend remains unchanged. Layering types B and G have the lowest *R* ^2^, while type J shows the highest *R* ^2^. Other dust-related layering types, such as E and F, also demonstrate strong *R* ^2^ values.

In fact, additional factors may impact the retrieval accuracy in the EM region, and also in general, in other parts of the world: variations in wind speed, humidity, and temperature inversions can alter aerosol dispersion and stratification; the interplay of multiple aerosol layers (e.g., lower marine layers overlain by dust) creates complex optical interactions; differences in scattering, absorption, and refractive indices of aerosols originating from different sources (North African dust vs Middle Eastern dust); long-range transport pathways that affect aerosol age, composition, and distribution. Again, MAIAC’s assumptions about aerosol types and their vertical distribution may not fully capture the characteristics of aerosols for nondust-dominated profiles.

Similar patterns emerged when comparing AERONET and CALIPSO AOD, where the best fits were obtained under dust-dominated conditions.^96^ These findings suggest that current MAIAC algorithms need refinement to improve accuracy in multilayered nondust profiles, ensuring reliable performance across diverse atmospheric conditions.

### 3.5 Comparing Lidar, Ground-Based, and Satellite Observations in Pollution Monitoring

The correlation heat maps in Figure 7 show the relationships between lidar (0–1000 m and 1000–3000 m) and nonlidar parameters for each layering type. Layering Types E and I had the highest number of significant associations (*N* = 21 and 23 respectively, *p* < 0.05), whereas Types A and D had the lowest number (4 and 7, respectively). A high number of significant associations might indicate different dependencies between aerosol properties, hinting at the complexity of pollution dynamics. In contrast, a low number of associations can highlight isolated or weakly correlated factors, suggesting event-specific pollution. Future studies shall be conducted to better understand these interactions for different pollution scenarios.

**Figure 7:**
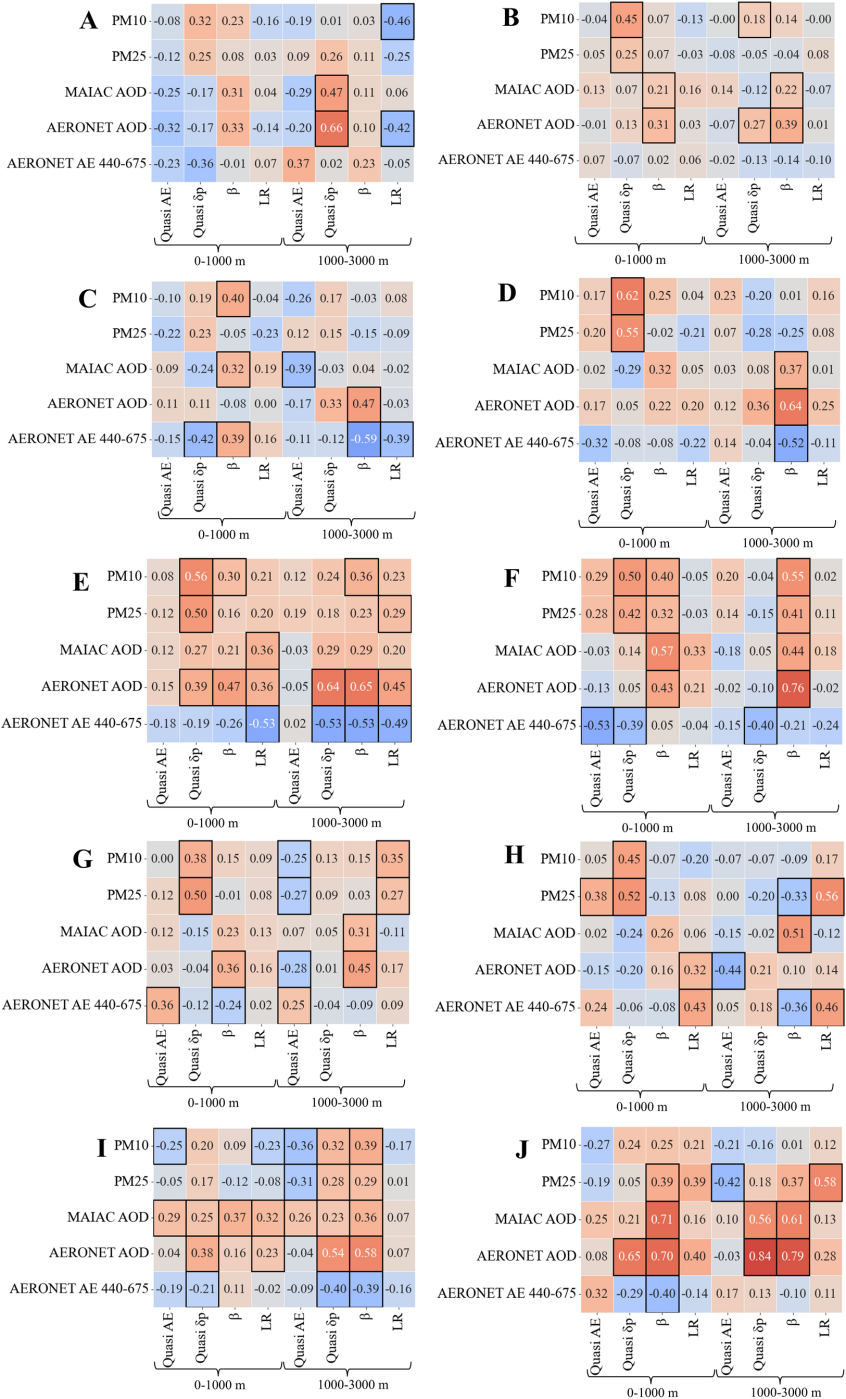
Correlation heat maps for each layering type (A–J) showing the relationships between different lidar (quasi AE, quasi *δp*, β, lidar ratio) and ground/satellite (*PM*_10_, *PM*_25_, MAIAC AOD, AERONET AOD, and *AE*_440–675_) measurements for two height ranges: 0–1000 m, and 1000–3000 m. Color scale indicates the strength and direction of the correlations, with red representing positive correlations and blue negative ones. Black outlines highlight significant correlations (*p* < 0.05).

On average (Figure S3), the relationship between AERONET AE and quasi *δp* was significant at all altitudes and became stronger with height (−0.47, and −0.52 for 0–1000 m and 1000–3000 m, respectively). The increasing association with altitude likely reflects more homogeneous aerosol layers at higher altitudes, compared to the lower atmosphere, which is influenced by local pollution sources and sea breeze effects. The strong negative correlation aligns with expectations (i.e., anthropogenic aerosols exhibit high AE and low quasi *δp*). The relationship between *PM*_10_/*PM*_2.5_, and quasi *δp* decreased with height, with *r* = 0.69, and 0.33 for *PM*_2.5_ at 0–1000 m, and 1000–3000 m, respectively. As expected, the strongest correlation was at ground level, as lofted dust layers are not reflected in surface *PM*_10_ concentrations.^13^ These findings align with Bellini et al.^97^ who reported that *PM*_10_ can be predicted using lidar, albeit with a 35% discrepancy.

#### 3.5.1 Dust Prevalent Conditions vs Lidar Correspondence

The optical properties of Middle Eastern dust differ from those of African dust.^98,99^ Indeed, these layering types had different lidar vs nonlidar measurement correspondences. Type J (Middle East dust source: dust layer with overlying anthropogenic component) exhibited the strongest correlations between MAIAC AOD and β (*r* = 0.71 at 0–1000 m and *r* = 0.61 at 1000–3000 m), and a relatively strong association with quasi *δp* at 1000–3000 m (*r* = 0.56). These results align well with the MAIAC retrieval definitions that are optimized for the presumably prevalent conditions in the EM region.^55^ In contrast, Type E (North African dust) showed a much weaker correspondence between MAIAC AOD and lidar-derived parameters, as it is not fully represented in the retrieval’s classification lookup table. AERONET AOD for Type E exhibited significant associations with most lidar-derived measurements (0.34 < *r* < 0.65), along with a good correspondence between *PM*_10_ and quasi *δp* (at 0–300 m): *r* = 0.72 (*r* = 0.63 for *PM*_2.5_). When anthropogenic pollution is mixed with dust, there is a higher correspondence between lidar-derived and *PM* concentrations measured at air quality stations. For example, Type D (marine layer overlain by anthropogenic and dust mix): *r* = 0.62 for *PM*_10_ and *r* = 0.55 for *PM*_2.5_ (at 0–1000 m). The direct implication of our results is the possibility of replacing ground-based measurements with lidar-related ones during dust events.

#### 3.5.2 Anthropogenic Conditions

For prevalent anthropogenic conditions (A, B, G), only Type A showed a significant, but much lower correlation between MAIAC and quasi *δp* (*r* = 0.47), with no correspondence between ground-based *PM*_2.5_/*PM*_10_ concentrations and lidar parameters. The weak correlation between MAIAC AOD and quasi *δp* for anthropogenic-dominated types (A, B, G), and the absence of any correspondence with ground-based *PM*_2.5_/*PM*_10_ concentrations, underscore the challenges in capturing the complexity of anthropogenic pollution layers using current satellite and lidar methodologies. These findings have significant implications for health-related studies, where satellite-derived AOD is often linked to ground-level PM concentrations to estimate exposure. Without a clear relationship between these variables, exposure assessments may lack accuracy, especially under conditions of vertical stratification.

### 3.6 Feasibility Study as an Example of Future Applications: Estimation of Vertical Layering Type and Additional Considerations

Figure 8 presents a preliminary classification report and a confusion matrix of the true pollution types and those predicted by the RF algorithm. The model achieved an overall accuracy of 61%. The highest precision was observed for Type J (86%, Middle Eastern dust with lofted anthropogenic layer) and Type C (85%, anthropogenic first layer with lofted dust layer), whereas the model was unable to predict Type D (marine layer overlain by anthropogenic + dust layer). Most of the Type D days were misclassified as Type B (marine layer with overlying anthropogenic layer).

**Figure 8:**
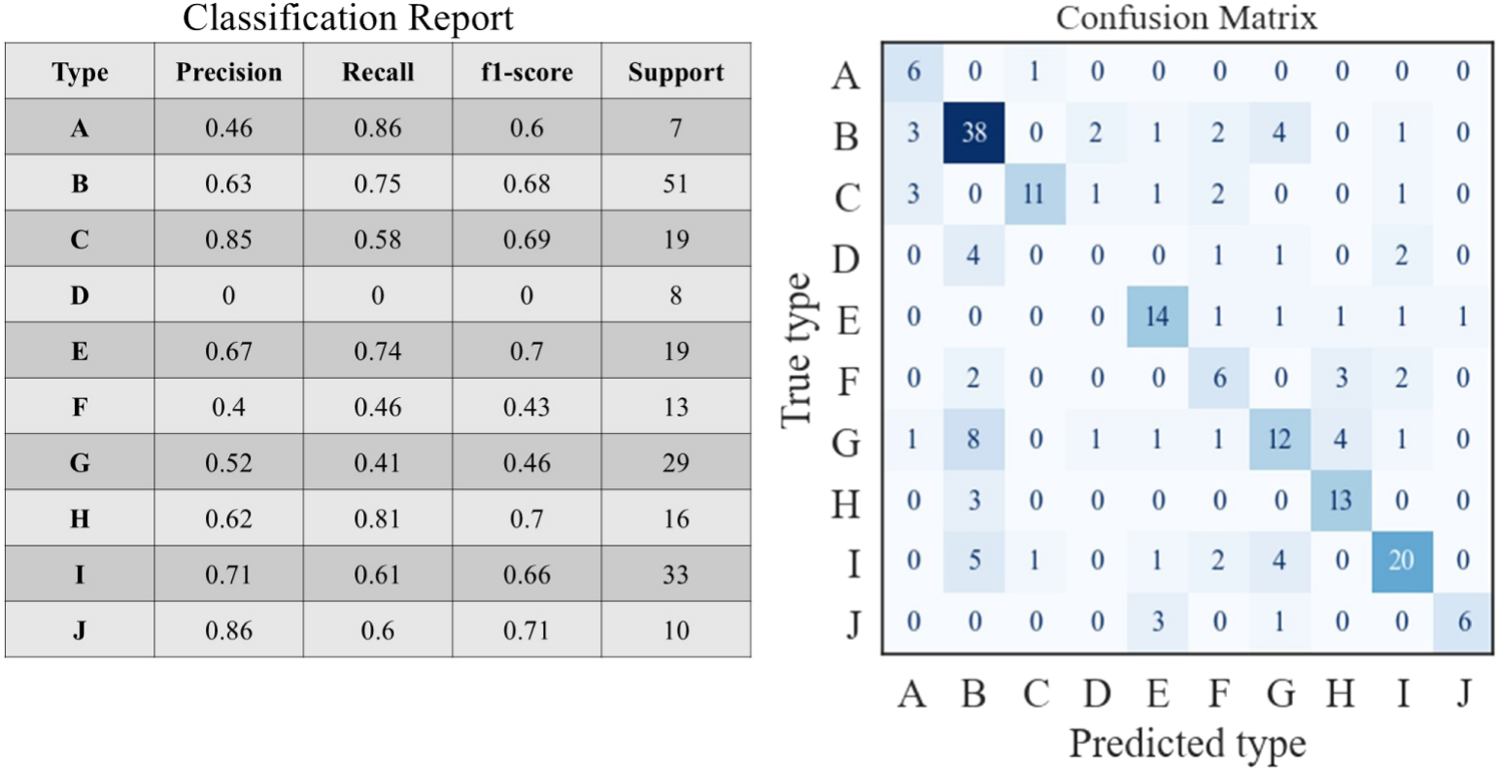
Classification report and confusion matrix displaying the RF algorithm-predicted and true classification results for the different layering types. Color intensity represents the number of classifications, with darker shades indicating higher counts.

Similarly, Type G (with 52% precision) shares characteristics with Type B, both involving a marine first layer and anthropogenic upper layer, although Type G has a marine impact in the upper layer. This led to 28% of Type G days being predicted as Type B, further illustrating the model’s difficulty in differentiating between types with overlapping features.

Despite these challenges, the model performed well in classifying Types A, B, C, E, H, I, and J, which had relatively high precision and recall. Encouraging results have also been achieved by Kalantari et al.^100^ who aimed to predict air quality index (eight categories) using artificial intelligence (AI) models. Those authors’ achieved accuracies ranging between 0.6 and 0.86, with different models considered.

Our preliminary results guide us on several additional important strategies that should be employed in future studies:

- Increasing sample size, thereby enlarging underrepresented classes, which could help the model better learn these categories and reduce misclassification rates.
- Identifying and incorporating additional parameters and contextual information that can better distinguish between similar types.^101^ For example, meteorology and synoptic classification,^43^ along with additional aerosol optical parameters, could improve the model’s ability to differentiate between overlapping categories. Note that this was not the main goal of the present study.

Importantly, the pollution types presented in our study can be used in more advanced AI models as training data sets.

## 4 Further Directions of Research

Our study integrates ground-based lidar profiling, satellite aerosol retrievals, and back-trajectory modeling to systematically classify and assess pollution layering. While we identified 10 distinct pollution types at a single site, understanding broader spatial variability remains a challenge.^17,58,102^ Expanding this research requires integrating continuous and extensive data sets, such as CALIPSO and EarthCARE satellite observations or ground-based networks like ACTRIS (Aerosol, Clouds, and Trace Gases Research Infrastructure).^103^ These additional data sources would help capture the heterogeneity of pollution layers across different regions.^104–109^

Pollution layering affects radiation balance, cloud properties, and atmospheric heating rates, influencing temperature and precipitation trends.^110^ As climate projections suggest an increasing dominance of the Persian Trough in the coming years,^111^ the frequency of summer-type pollution layers, primarily associated with anthropogenic emissions, is expected to rise. Future research should focus on assessing the implications of this trend and guiding environmental policy measures to mitigate the resulting air quality challenges.

Long-range pollution transport significantly shapes regional air quality. Trajectory analyses provide valuable insights into pollution sources and transport pathways but are limited by reliance on modeled meteorology and the exclusion of chemical transformations during transport. Future studies should integrate high-resolution atmospheric simulations, such as WRF (Weather Research and Forecasting),^76^ with diurnal lidar observations to better capture meteorological processes affecting pollution dispersion. This combined approach would improve air quality assessments by linking wind patterns, local emissions, and urban morphology.^112^

Finally, integrating vertical aerosol layering conditions into satellite AOD retrievals could help reduce existing biases in pollution estimates. Additionally, incorporating this data set into health studies would improve assessments of pollution exposure and its potential health impacts. For example, distinguishing between locally emitted traffic pollution near the surface and transported fine particulate matter at higher altitudes can improve air quality assessments and health impact studies.

## Supplementary Material



## Notes

The study area map (Figure S1); Aerosol layering classification decision tree (Figure S2); Correlation heatmap showing the relationships between different Lidar and ground/satellite measurements (Figure S3); Average and standard deviation of ground-based and Lidar measurements of each layering type (Table S1) (PDF)
