# Supplementary material for: Unveiling Atmospheric Layers: Vertical Pollution Patterns and Prospects for High-Resolution Aerosol Retrievals Using the Eastern Mediterranean as a Case Study
Source: Environ Sci Technol. 2025 Jun 10;59(24):12181–95. doi: 10.1021/acs.est.4c14556 (PMC12199465; doi:10.1021/acs.est.4c14556)
Supplement: Supplementary file 1 [file es4c14556_si_001.pdf]

## Supporting Information for:

# Unveiling Atmospheric Layers: Vertical Pollution Patterns and Prospects for High-Resolution Aerosol Retrievals Using the Eastern Mediterranean as a Case Study

**Irina Rogozovsky**[1], **Albert Ansmann**[2], **Julian Hofer**[2], **Alexandra Chudnovsky**[3]

[1]Porter School of the Environment and Earth Sciences, Raymond and Beverly Sackler Faculty of Exact Sciences, Tel Aviv University, Tel Aviv, 6997801, Israel

[2]Leibniz Institute for Tropospheric Research, Leipzig, 04318, Germany

[3]Department of Geophysics, Raymond and Beverly Sackler Faculty of Exact Sciences, Tel Aviv University, Tel Aviv, 6997801, Israel

**Corresponding Authors email address:** irinar@mail.tau.ac.il, achudnov@tauex.tau.ac.il

**Summary:** 3 pages including 3 figures and 1 table.

### Table of Contents

- Figure S1: .......... S1
- Figure S2: .......... S2
- Figure S3: .......... S2
- Table S1: .......... S3

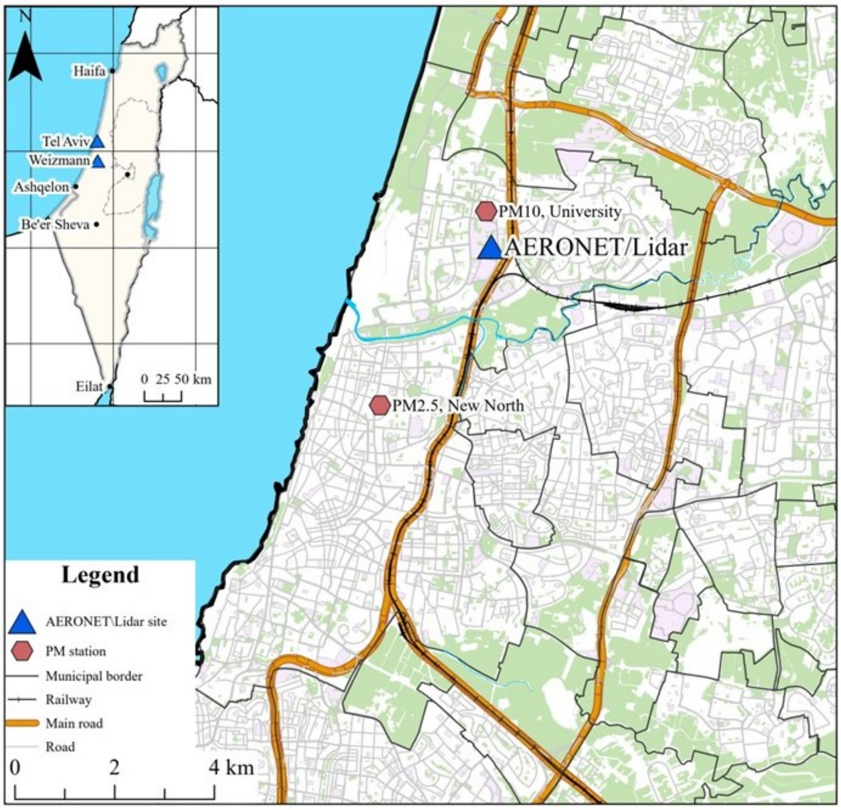


Fig. S1: Map of the study area showing the locations of ground monitoring stations (hexagons) and Lidar and AERONET stations (blue triangles).

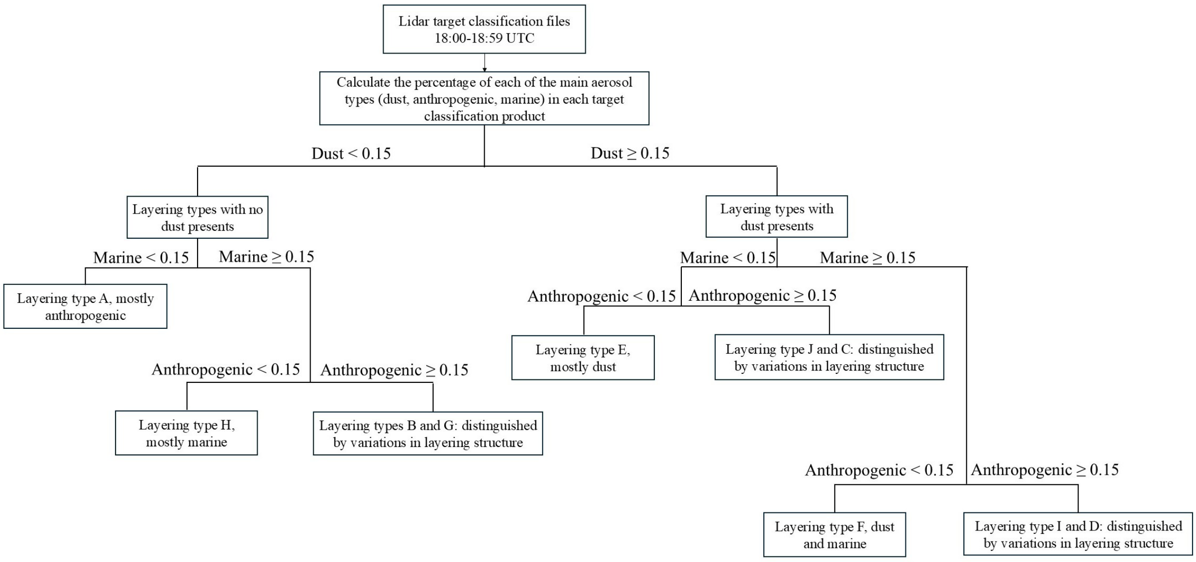


Fig. S2: Decision tree of classification of aerosol layering types based on lidar target classification product. The decision tree categorizes layering types according to dust, marine, and anthropogenic aerosol contributions, further distinguishing specific types based on their structural variations.

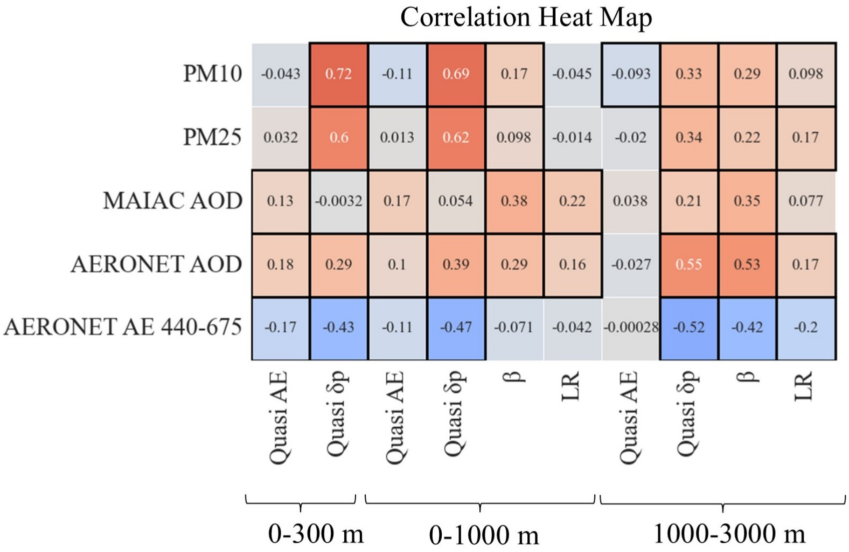


Fig. S3: Correlation heatmap showing the relationships between different Lidar (quasi AE, quasi $\delta_p$, $\beta$, LR) and ground/satellite measurements ($PM_{10}$, $PM_{25}$, MAIAC AOD, AERONET AOD, AERONET $AE_{440-675}$) at three height ranges: 0-300 m, 0-1000 m, and 1000-3000 m. The color scale indicates the strength and direction of the correlations, with red representing positive correlations and blue representing negative correlations. Black outlines highlight significant correlations ($p < 0.05$).

| | Lidar 0-1000 m | | | | Lidar 1000-3000m | | | | Environmental stations | | MAIAC | AERONET | |
|---|---|---|---|---|---|---|---|---|---|---|---|---|---|
| **Type** | **Quasi AE 532-1064 nm** | **Quasi δp 532 nm** | **β 532 nm** | **LR 532 nm** | **Quasi AE 532-1064 nm** | **Quasi δp 532 nm** | **β 532 nm** | **LR 532 nm** | **$PM_{10}$** | **$PM_{2.5}$** | **AOD 470 nm** | **AOD 470 nm** | **AE 440-675 nm** |
| **A** | 1.34 ± 0.28 | 0.03 ± 0.01 | 1.18e-06 ± 6.93e-07 | 29.04 ± 13.28 | 1.42 ± 0.33 | 0.05 ± 0.06 | 6.42e-07 ± 4.28e-07 | 37.91 ± 8.51 | 22.24 ± 8.88 | 12.48 ± 5.24 | 0.28 ± 0.14 | 0.21 ± 0.10 | 1.21 ± 0.36 |
| **B** | 0.91 ± 0.22 | 0.03 ± 0.01 | 1.04e-06 ± 6.60e-07 | 28.80 ± 12.18 | 1.25 ± 0.17 | 0.03 ± 0.03 | 5.37e-07 ± 4.45e-07 | 34.62 ± 7.93 | 23.97 ± 12.68 | 12.74 ± 5.94 | 0.24 ± 0.10 | 0.17 ± 0.07 | 1.26 ± 0.24 |
| **C** | 1.38 ± 0.28 | 0.05 ± 0.02 | 1.27e-06 ± 8.79e-07 | 31.46 ± 13.24 | 1.36 ± 0.38 | 0.10 ± 0.06 | 1.19e-06 ± 8.79e-07 | 37.13 ± 9.22 | 26.17 ± 9.74 | 15.40 ± 5.35 | 0.31 ± 0.18 | 0.28 ± 0.12 | 0.83 ± 0.40 |
| **D** | 0.80 ± 0.19 | 0.05 ± 0.02 | 8.69e-07 ± 5.58e-07 | 22.73 ± 10.82 | 1.19 ± 0.23 | 0.05 ± 0.05 | 7.16e-07 ± 8.56e-07 | 32.63 ± 8.14 | 24.17 ± 11.78 | 12.39 ± 6.17 | 0.22 ± 0.11 | 0.18 ± 0.09 | 1.02 ± 0.34 |
| **E** | 0.79 ± 0.36 | 0.14 ± 0.04 | 1.29e-06 ± 8.06e-07 | 28.13 ± 11.54 | 1.07 ± 0.42 | 0.13 ± 0.06 | 1.48e-06 ± 9.45e-07 | 37.26 ± 9.49 | 67.79 ± 43.88 | 24.94 ± 13.28 | 0.29 ± 0.16 | 0.33 ± 0.18 | 0.46 ± 0.33 |
| **F** | 0.56 ± 0.21 | 0.06 ± 0.03 | 1.08e-06 ± 7.64e-07 | 27.46 ± 11.38 | 0.83 ± 0.26 | 0.09 ± 0.05 | 1.12e-06 ± 9.04e-07 | 34.07 ± 8.38 | 37.56 ± 45.08 | 16.36 ± 14.09 | 0.29 ± 0.16 | 0.23 ± 0.16 | 0.86 ± 0.38 |
| **G** | 0.67 ± 0.17 | 0.03 ± 0.02 | 1.20e-06 ± 6.89e-07 | 27.99 ± 11.74 | 1.01 ± 0.19 | 0.03 ± 0.03 | 6.84e-07 ± 6.51e-07 | 33.48 ± 9.14 | 26.00 ± 13.94 | 12.38 ± 5.19 | 0.25 ± 0.10 | 0.18 ± 0.09 | 1.26 ± 0.29 |
| **H** | 0.38 ± 0.19 | 0.03 ± 0.02 | 9.66e-07 ± 6.77e-07 | 26.10 ± 11.23 | 0.69 ± 0.28 | 0.03 ± 0.02 | 1.16e-06 ± 1.19e-06 | 27.47 ± 6.85 | 23.47 ± 9.03 | 10.44 ± 4.93 | 0.23 ± 0.11 | 0.17 ± 0.09 | 1.17 ± 0.31 |
| **I** | 0.99 ± 0.32 | 0.03 ± 0.01 | 1.35e-06 ± 7.41e-07 | 33.90 ± 14.62 | 1.21 ± 0.27 | 0.07 ± 0.04 | 9.08e-07 ± 6.92e-07 | 37.36 ± 6.36 | 20.65 ± 12.72 | 11.43 ± 5.47 | 0.30 ± 0.12 | 0.22 ± 0.11 | 0.84 ± 0.31 |
| **J** | 0.65 ± 0.17 | 0.11 ± 0.04 | 1.14e-06 ± 1.07e-06 | 26.45 ± 5.81 | 1.01 ± 0.25 | 0.08 ± 0.06 | 1.12e-06 ± 1.13e-06 | 35.89 ± 5.68 | 66.51 ± 43.73 | 20.88 ± 8.84 | 0.28 ± 0.24 | 0.22 ± 0.15 | 0.92 ± 0.31 |

Table S1: Summarize the average and standard deviation for all the characteristics of each layering type.
